# Derivate Isocorydine (d-ICD) Suppresses Migration and Invasion of Hepatocellular Carcinoma Cell by Downregulating *ITGA1* Expression

**DOI:** 10.3390/ijms18030514

**Published:** 2017-02-27

**Authors:** Xiaoqin Liu, Hua Tian, Hong Li, Chao Ge, Fangyu Zhao, Ming Yao, Jinjun Li

**Affiliations:** State Key Laboratory of Oncogenes and Related Genes, Shanghai Cancer Institute, Renji Hospital, Shanghai Jiaotong University School of Medicine, 25/Ln 2200, Xietu Road, Shanghai 200032, China; liuxiaoqin106484@163.com (X.L.); htian@shsci.org (H.T.); hongli@shsci.org (H.L.); chaoge1127@163.com (C.G.); fangyuzhao11@163.com (F.Z.); myao@shsci.org (M.Y.)

**Keywords:** d-ICD, *ITGA1*, migration, invasion, hepatocellular carcinoma

## Abstract

In our previous studies, we found that isocorydine (ICD) could be a potential antitumor agent in hepatocellular carcinoma (HCC). Derivate isocorydine (d-ICD), a more effective antitumor agent, has been demonstrated to inhibit proliferation and drug resistance in HCC. In order to investigate the potential role of d-ICD on HCC cell migration and its possible mechanism, wound healing assay, trans-well invasion assay, western blot analysis, and qRT-PCR were performed to study the migration and invasion ability of HCC cells as well as relevant molecular alteration following d-ICD treatment. Results indicated that the migration and invasion ability of HCC cells were suppressed when cultured with d-ICD. Meanwhile, the expression level of *ITGA1* was markedly reduced. Furthermore, we found that *ITGA1* promotes HCC cell migration and invasion in vitro, and that *ITGA1* can partly reverse the effect of d-ICD-induced migration and invasion suppression in HCC cells. In addition, dual luciferase reporter assay and chromatin immunoprecipitation assay were used to study the expression regulation of *ITGA1*, and found that *E2F1* directly upregulates *ITGA1* expression and d-ICD inhibits *E2F1* expression. Taken together, these results reveal that d-ICD inhibits HCC cell migration and invasion may partly by downregulating *E2F1*/*ITGA1* expression.

## 1. Introduction

Liver cancer is the second leading cause of cancer death worldwide, and most (70% to 90%) pathological types of liver cancers are hepatocellular carcinoma (HCC) [1]. The main cause of cure difficulty in HCC patients is the lack of effective drugs for tumor recurrence and metastasis [2]. Thus, the need for developing an effective antineoplastic for metastatic HCC is extremely urgent.

Isocorydine (ICD), as an alkaloid, is purified from *Papaveraceae* sp. plants, such as *Dactylicapnos scandens Hutchins* and *Dicranostigma leptopodum* (Maxim.) *Fedde*. It is used as a clinic drug for the treatment of spasmolytic, vasodilating, antiplasmodial, and antiarrhythmic symptoms. Our previous studies demonstrated that ICD inhibited HCC growth by inducing cell cycle arrest and apoptosis of HCC cells [3], and significantly decreasing the percentage of side population (SP) cells and sensitizing cancer cells to chemical therapy in HCC cell lines [4]. However, the effective dosage of ICD for anti-HCC reached up to 200 μM, which has been proposed to be too high a dosage for clinical treatment. Derivate isocorydine (d-ICD), the chemical structure of ICD modified at C-8 (8-amino-isocorydine) [4,5], has proved to be more stable and effective regarding anticancer activity [5]. Our previous studies showed that d-ICD suppressed HCC cell growth both in vitro and in vivo and inhibited drug resistance at a lower dosage [6,7]. However, whether or not the more effective anti-HCC agent d-ICD has an effect on the migration and invasion of HCC cells is still unknown. In this study, we investigated the effects of d-ICD on HCC cell migration and invasion, as well as the underlying molecular mechanism.

Integrin-mediated adhesion of cell-to-cell and cell-to-extracellular matrix and integrin dysregulation have been proposed to partly contribute to tumor progression and metastasis [8,9,10,11]. As one of the integrin family members, integrin alpha1, which is encoded by the *ITGA1* gene, was first investigated in immune disease [12]. Further studies have suggested that integrin alpha1 is closely associated with angiogenesis [13,14]. Recent studies have revealed that *ITGA1* is upregulated in higher metastasis potential melanoma cells, gastric cancer cells, and colorectal cancer cells [15,16,17]. Blocking integrin α1 with a therapeutic monoclonal antibody or knocking out *ITGA1* in mice both indicated that *ITGA1* promoted tumor cell migration and invasion [18,19,20]. However, the specific role of *ITGA1* in HCC has not been fully understood.

*E2F1* is a well-known transcriptional factor that widely regulates gene expression that is involved in proliferation, autophagy, and apoptosis [21,22]. The biological function of *E2F1* has been shown to be controversial during HCC tumorigenesis [23,24]. In our present study, we found that *E2F1* may also upregulate *ITGA1* expression in HCC.

In the present study, we found that d-ICD treatment inhibited HCC cell migration and invasion in vitro, and that inhibition was partly achieved by downregulating *E2F1*/*ITGA1* expression. Taken together, our results further proved that d-ICD may be a more effective anti-tumor agent in HCC.

## 2. Results

### 2.1. d-ICD Inhibits HCC Cell Migration and Invasion In Vitro and Downregulates ITGA1 Expression

**Figure 1:**
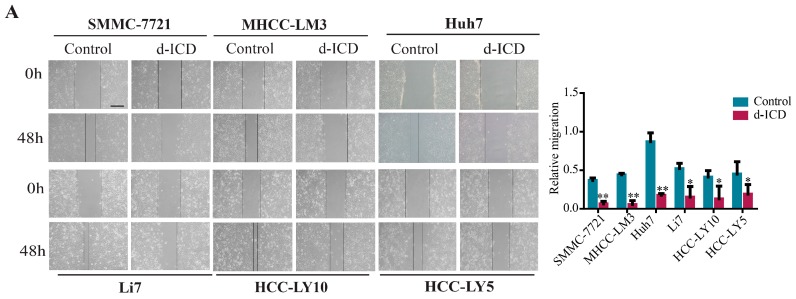

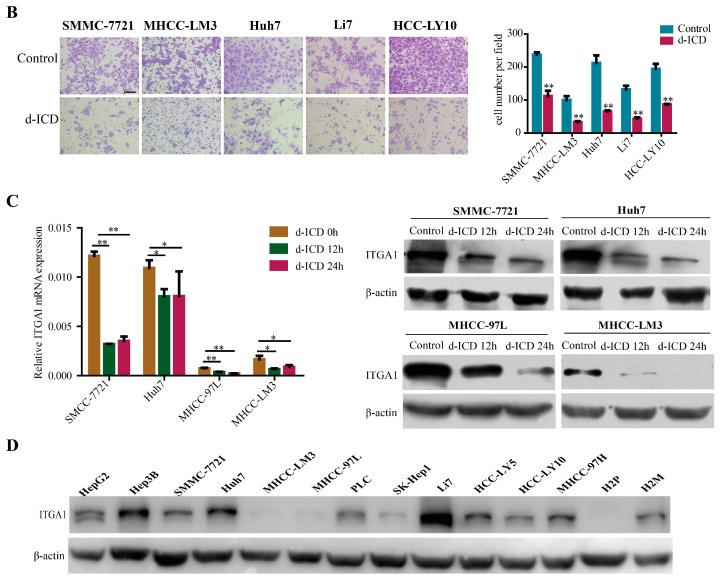
Derivate isocorydine (d-ICD) inhibits hepatocellular carcinoma (HCC) cell migration and invasion and *ITGA1* expression. (**A**) Various HCC cell lines were incubated with d-ICD, and the wound-healing assay was performed to analyze the migration ability (scale bar stand for 200 μm); (**B**) The Trans-well assay was performed to analyze the invasion ability of HCC cells after d-ICD treatment (scale bar stand for 100 μm); (**C**) RT-PCR and western blotting analyses were performed to examine *ITGA1* expression in HCC cells with d-ICD treatment for 0 h, 12 h, and 24 h; (**D**) Western blot analyzed endogenous *ITGA1* expression in HCC cells. (* *p* < 0.05, ** *p* < 0.01).

We performed the wound healing assay and trans-well assay with or without d-ICD treatment in HCC cells. Different types of HCC cell lines vary in sensitivity to d-ICD and the d-ICD concentration according to a previous study in our laboratory [6,7]. The results showed that d-ICD inhibited HCC cell migration and invasion (Figure 1A,B). To further elucidate the molecular mechanism involved in this phenomenon, we analyzed the differential gene expression obtained from the cDNA microarrays of HCC cell lines SMMC-7721 and Huh7 between the d-ICD treated HCC cells and control group obtained from a previously published study [7]. Combined with the fold changes and biological function of the differentially expressed genes, we finally targeted against *ITGA1* and confirmed the downregulation of *ITGA1* in HCC cell lines Huh7, SMMC-7721, MHCC-97L, and MHCC-LM3 via qRT-PCR and western blotting analyses (Figure 1C). Next, we analyzed the intrinsic *ITGA1* expression in HCC cell lines using qRT-PCR and western blotting analyses (Figure 1D and Figure S1).

### 2.2. Overexpression of ITGA1 Promotes HCC Cell Migration and Invasion In Vitro

**Figure 2:**
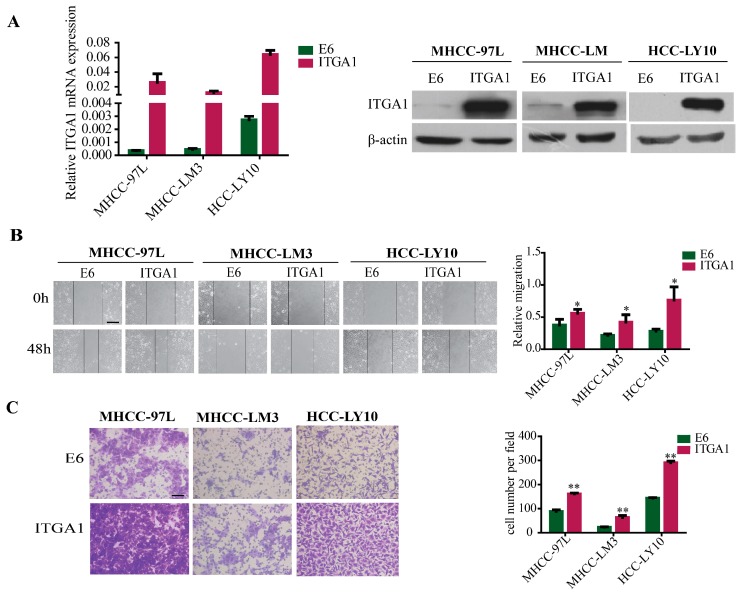
Overexpression of *ITGA1* promotes HCC cell migration and invasion in vitro. (**A**) The efficiency of *ITGA1* overexpression were analyzed via RT-PCR and western blot; (**B**) The in vitro migration ability of MHCC-97L, MHCC-LM3 and HCC-LY10 cells stably transfected with *ITGA1* or vector were assessed using the wound-healing assay (scale bar stand for 200 μm); (**C**) The in vitro invasion ability of those *ITGA1*-overexpressed HCC cells were assessed using the trans-well assay (scale bar stand for 100 μm). (*p < 0.05, ** *p* < 0.01).

To further investigated the role of *ITGA1* in HCC, we selected the HCC cell lines MHCC-97L, MHCC-LM3, and HCC-LY10, which show intrinsically lower expression of *ITGA1*, to reconstruct *ITGA1* stably overexpression in HCC cells by using *ITGA1* lentiviral infection and control lentivirus EX-NEG, also known as E6. The overexpression efficiency was confirmed using qRT-PCR and western blotting analyses (Figure 2A). We examined the function of *ITGA1* on HCC cells, and the results of the wound healing assay and trans-well invasion assay demonstrated that overexpression of *ITGA1* promoted HCC cell migration and invasion in vitro (Figure 2B,C).

### 2.3. Silencing ITGA1 Expression Inhibits HCC Cell Migration and Invasion In Vitro

**Figure 3:**
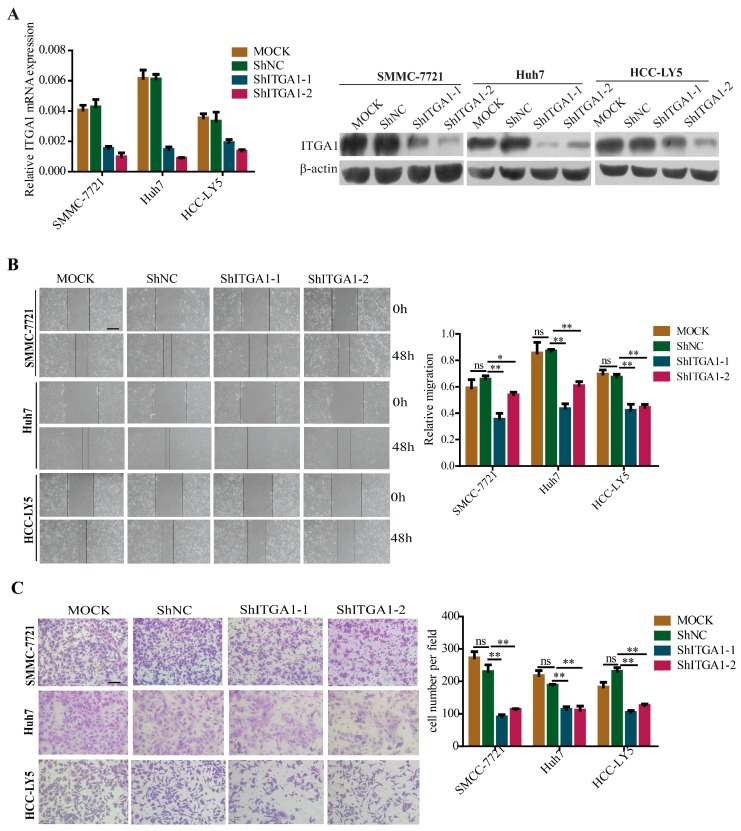
Silencing *ITGA1* expression inhibits HCC cell migration and invasion in vitro. (**A**) qRT-PCR and western blot analyzed the silencing efficiency of *ITGA1* expression; (**B**) The in vitro migration ability of SMMC-7721, Huh7 and HCC-LY5 cells stably transfected with *ITGA1* ShRNA or ShNC or MOCK cells were assessed using wound-healing assay (scale bar stand for 200 μm); (**C**) The in vitro invasion ability of those *ITGA1*-silencing HCC cells were assessed by trans-well assay (scale bar stand for 100 μm). (“ns” indicates no statistical significance, * *p* < 0.05, ** *p* < 0.01).

Conversely, we selected the HCC cell lines SMMC-7721, Huh7, and HCC-LY5, which intrinsically express higher levels of *ITGA1* to knock down endogenous *ITGA1* expression via shRNA and control lentiviral infection. The *ITGA1* silencing efficiency was confirmed by qRT-PCR and western blotting analyses (Figure 3A). Similarly, we performed basic function assays to analyze the *ITGA1* function in HCC cells and found that *ITGA1* knockdown inhibited HCC cell migration and invasion (Figure 3B,C). Furthermore, to study whether the expression of *ITGA1* affecting the expression of ITGB1, we detected ITGB1 expression and found that there was no change in the expression of ITGB1 in *ITGA1*-overexpressed or *ITGA1*-silenced expression of HCC cells (Figure S2), which was consistent with a previous report by Pozzi A [25].

### 2.4. Overexpression of ITGA1 Partly Rescues d-ICD-Induced Migration and Invasion Inhibition in HCC Cells

**Figure 4:**
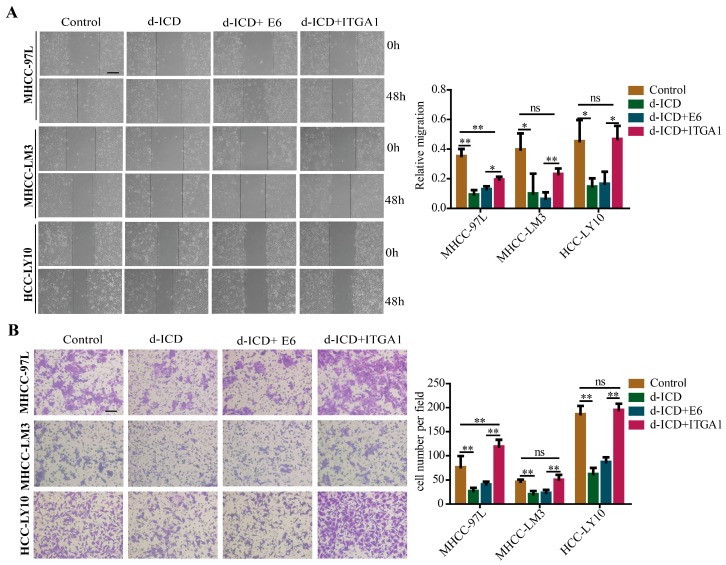
*ITGA1* partly rescues d-ICD-induced migration and invasion suppression in HCC cells; (**A**) The wound-healing assay was performed to detect the migration ability of MHCC-97L, MHCC-LM3 and HCC-LY10 empty cells after treating with or without d-ICD, and MHCC-97L, MHCC-LM3, and HCC-LY10 stably transfected with *ITGA1* or vector after treating with d-ICD (scale bar stand for 200 μm); (**B**) The Trans-well assay was performed to detect the invasion ability of those HCC cells (scale bar stand for 100 μm). (“ns” indicates no statistical significance, * *p* < 0.05, ** *p* < 0.01).

To determine whether the inhibition of HCC migration and invasion by d-ICD could be dependent on its inhibition on *ITGA1* expression, we performed functional compensation assays (Figure 4A,B). Similar results were observed that following d-ICD treatment, the migration and invasion ability of HCC cells was decreased. *ITGA1*-overexpressed HCC cells moved and invaded faster than E6 control group HCC cells following d-ICD treatment. The migration and invasion ability of HCC cells with *ITGA1* overexpression and d-ICD treatment showed no obvious difference when compared with empty HCC cells, suggesting that *ITGA1* overexpression partially abrogated the inhibition of d-ICD on migration and invasion of HCC cells. On the basis of previous results, we concluded that d-ICD inhibits HCC cell migration and invasion partly by downregulating *ITGA1* expression.

### 2.5. E2F1 Upregulates ITGA1 Expression and d-ICD Inhibits E2F1 Expression in HCC Cells

**Figure 5:**
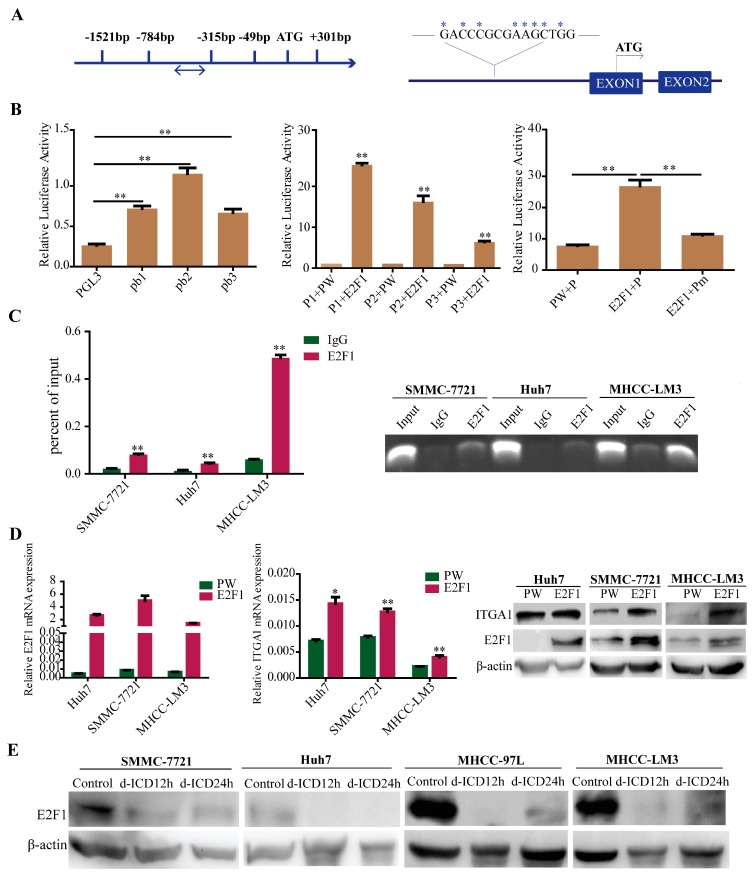
*E2F1* transcriptional upregulates *ITGA1* expression and d-ICD inhibits *E2F1* expression in HCC cells. (**A**) Identification of potential *E2F1* binding sites (double arrow marked) in the *ITGA1* promoter (arrow direction indicates sequence from 5′ to 3′) according to four TF-binding prediction websites (QIAGEN, ALGGEN PROMO, TFBIND, GENE REGULATION), and the mutant sites are marked (arrow direction indicates transcriptional direction of the downstreem gene); (**B**) Dual luciferase reporter gene studies were performed to detect the *ITGA1* promoter and its truncated and mutant construct activity in 293T cells; (**C**) Binding of *E2F1* to the *ITGA1* promoter in HCC cells was analyzed by chromatin immunoprecipitation. qRT-PCR was used to analyze the *ITGA1* promoter, and agarose gel electrophoresis was used to analyze the crosslinking status; (**D**) qRT-PCR and western blotting analyzed *E2F1* and *ITGA1* expression in HCC cells with transiently transfected *E2F1* plasmid; (**E**) Western blotting analyses of *E2F1* expression in HCC cells treated with d-ICD at different time points. (* *p* < 0.05, ** *p* < 0.01).

To further study the regulation of *ITGA1* in HCC cells, the *ITGA1* promoter was analyzed via transcriptional factors binding predicted websites TFBIND, ALGGEN-PROMO, GENE REGULATION, and QIAGEN. All of the four websites predicted that the *E2F1* binding site was located at −330 bp/−315 bp of the *ITGA1* promoter (Figure 5A). We further found that the expression of *ITGA1* was upregulated in *E2F1* overexpression HCC cells (Figure 5D), and was downregulated in *E2F1* silencing HCC cells (Figure S3). Meanwhile, we observed that the expression of *ITGA1* positively related to the expression of *E2F1* in HCC tissue (Figure S4). Based on the prediction and expression analysis of *E2F1* and *ITGA1*, we hypothesized that *E2F1* may bind to the *ITGA1* promoter and regulate *ITGA1* expression. To confirm our hypothesis, we constructed clones of the *ITGA1* promoter as well as truncated and mutant variations. Dual luciferase reporter gene studies were performed to detect the activity of the *ITGA1* promoter in 293T cells (Figure 5B), and in HCC cells (Figure S5). These results revealed that *E2F1* increased *ITGA1* promoter activity. However, *E2F1* did not increase *ITGA1* promoter activity containing a putative *E2F1*-mutated binding site (Figure 5B). Binding of *E2F1* to the *ITGA1* promoter was further confirmed using a chromatin immunoprecipitation assay (Figure 5C). Meanwhile, we found that the expression of *E2F1* was downregulated after d-ICD treatment in HCC cells (Figure 5E), which prompted d-ICD suppression of HCC cell migration and invasion may partly via the *E2F1*/*ITGA1* pathway.

## 3. Discussion

It is well known that hepatocellular carcinoma greatly threatens human health due to the high morbidity and mortality and the cure difficulty. Thus, it is urgent to develop an effective drug for HCC treatment. In our previous studies, d-ICD has proven to be an effective anti-HCC agent due to its effects as an anti-proliferation and anti-drug-resistant agent [6,7]. In the present study, we continued to probe the function and relevant molecular mechanisms of d-ICD in HCC cells. We initially found that d-ICD inhibited HCC cell migration and invasion in vitro. Though d-ICD mildly induced HCC cell apoptosis during migration assay (Figure S6), but we still believe there must be another molecular mechanism during the process of d-ICD inhibiting HCC cell migration and invasion. So, we screened potential metastasis-related differential expression genes from the cDNA microarrays of Huh7 and SMMC-7721 cells between the control and d-ICD treatment groups [7], and due to our knowledge of its gene biological function, we selected the target gene *ITGA1*. Next, we cultured HCC cells with d-ICD and confirmed the decreased expression of *ITGA1* at the mRNA and protein levels.

Integrin α1 coupled with integrin β1 form a heterodimer located at the cell membrane [26]. Pozzi’s team has studied the function of integrin α1 in various diseases for many years and found that integrin α1 plays an important role in proliferation, adhesion, migration, and angiogenesis [26]. Many reports showed that *ITGA1* may play an important role on migration and invasion in various tumor [15,16,17,27]. In addition, Wan, et al found that overexpression of *ITGA1* in HCC cells presented high metastasis potential in the lymph gland [28]. To verify the migration and invasion inhibition of d-ICD to HCC cell partially by downregulating *ITGA1* expression, we further investigated the function of *ITGA1* in HCC cells. Consistent with the suggested role of *ITGA1* as a potential tumor promoter, we showed that the migration and invasion ability of HCC cells was enhanced when *ITGA1* was stably overexpressed, while the migration and invasion ability of HCC cells was decreased when *ITGA1* expression was stably knocked down. However, *ITGA1* has no obvious effect on HCC cell proliferation or apoptosis in vitro (Figure S7). The results of both overexpression and decreased expression of *ITGA1* indicated that *ITGA1* plays a critical role on HCC cell migration and invasion. Furthermore, the results of functional compensation experiments showed that *ITGA1* overexpression partially rescued the migration and invasion suppression of d-ICD in HCC cells. So far, based on these findings, we concluded that d-ICD inhibits HCC cell migration and invasion partly by down-regulating *ITGA1* expression.

Furthermore, we explored the expression regulation of *ITGA1* in HCC cells. Based on the prediction of the transcription factor website and our experimental results, we found that *E2F1* directly binds to the *ITGA1* promoter and that the expression of *ITGA1* positively correlated to the expression of *E2F1* in HCC cells and tissue, suggesting that *E2F1*, the powerful transcription factor, also transcriptionally upregulates *ITGA1* expression in HCC. There have been previous reports demonstrating that *E2F1* mediates tumor metastatic dissemination by up-regulating fibronectin [29]. In HCC, *E2F1* expression and its effects have been showed to be controversial during tumorigenesis [24,30,31]. Moreover, we found that *E2F1* is downregulated in HCC cells following d-ICD treatment, indicating that d-ICD inhibited *ITGA1* expression may partly through downregulating *E2F1* expression. These findings at least partially explains molecular mechanisms underlying action of d-ICD in inhibiting migration and invasion of HCC in vitro.

It is important that combination drugs to exert synergistic effect during routine chemotherapy. Isocorydine (ICD) has been reported to enhance the sensitivity of HCC cell lines to DXR by targeting HCC SP cells in our previous study [3]. Sorafenib, as an only one anti-HCC drug in clinics approved by FDA, was demonstrated to be more effective when combination administration with low dosage of d-ICD [2]. The function of d-ICD inhibiting HCC cells drug resistance may serve as the foundation of d-ICD being an adjuvant during anti-HCC chemotherapy. Therefore, we consider that d-ICD may improve the efficacy of many other chemotherapeutic drugs by enhancing the cytotoxic effect on HCC cells. Continuing studies are needed.

## 4. Materials and Methods

### 4.1. Cell Lines and Cell Culture

The human HCC cell lines SMMC-7721 was used in our study and were supplied by the Cell Bank of the Institute of Biochemistry and Cell Biology, China Academy of Sciences (Shanghai, China) in 2007. The cell lines MHCC-97L and MHCC-LM3 were kindly provided by the Liver Cancer Institute of Zhongshan Hospital, Fudan University (Shanghai, China) in 2007, and human HCC-LY5 and HCC-LY10 were established in our laboratory in 2011, which was authenticated by ourselves. 293T was purchased from the American Type Culture Collection (Manassas, VA, USA), and Huh7 cells were obtained from the Riken Cell Bank (Tsukuba, Japan) in 2010. The cell nutrient solution for all of the above cells was Dulbecco’s modified Eagle’s medium (DMEM) (Sigma-Aldrich, St. Louis, MO, USA) containing 10% fetal bovine serum (FBS) (HyClone), which was heat-inactivated at 56 °C for 30 min; 100 IU/mL penicillin G; and 100 μg/mL streptomycin (Sigma-Aldrich). All cell lines were incubated at 37 °C in a humidified atmosphere with 5% CO_2_. All cell lines used in this study were thawed fresh every two months and used within 20 passages. These cell lines were mycoplasma-free and authenticated by their examination of morphology and growth profile.

### 4.2. Western Blotting Analyses

Protein sample extraction and concentration detection were performed as previously described [6], and protein were separated onto 10% SDS-PAGE and transferred onto nitrocellulose or polyvinylidene difluoride (PVDF) membranes. Next, the membranes were blocked with 5% nonfat milk for 1 h at room temperature and incubated with primary antibodies (integrin α1 BAF 5676 R&D system, integrin β1 SC-59829, *E2F1* SC-251) overnight at 4 °C. After washing with 1× PBST 3 times, 10 min/each, a HRP-conjugated secondary antibody was added for 1 h at room temperature, and β-actin (A3854 Sigma-Aldrich) was used as a loading control. Chemiluminescence was detected using the Super Signal West Femto Chemiluminescent substrate kit (Thermo scientific, Waltham, MA, USA). Informed consent was obtained from all patients, and the study was approved by the Ethic Committee of Shanghai Jiao Tong University. Information on all of the antibodies used in this study is provided in Table S1.

### 4.3. Quantitative Real-Time Polymerase Chain Reaction (qRT-PCR)

Total RNA of cell lines was extracted using TRIzol reagent (Invitrogen, Carlsbad, CA, USA) and then reverse transcribed into cDNA using the PrimeScript™RT Reagent Kit (TaKaRa Biotechnology, Shimogyo-ku, Japan). Real-time PCR was performed using SYBR Premix Ex Taq (TaKaRa Biotechnology) according to the manufacturer’s protocol. The primers used to quantify the target genes or DNA fragments are provided in Table S2.

### 4.4. Plasmid Construction, Lentivirus Production, and Cell Transfection

Full-length human *ITGA1* plasmid and empty vector E6 were purchased from GeneCopoeia Company (Guangzhou, China), and the shRNA targeting *ITGA1* were designed by Genechem (Shanghai, China) and the sequence is provided in Table S3. The promoter sequences of *ITGA1* were cloned into PGL3-Basic (purchased from Promega, Madison, WI, USA) at the KpnI and HindIII sites. The full-length human *E2F1* gene coding sequence was PCR-amplified and cloned into pWPXL (Addgene, Cambridge, MA, USA) at the BamHI and EcoRI sites. The primers of the clones and promoter sequence are provided in Tables S4 and S5. Viral packaging and cell transfections were performed as previously described [32].

### 4.5. Wound Healing Assay

Approximately 1 × 10^6^ HCC cells were seeded onto 6-well plates, and after the cells were cultured overnight and overspread on the plates, we scratched a vertical long wound onto the cells and obtained images at 0 h and 48 h. Before we obtained the images, we washed the cells twice with 1× PBS and replaced the medium with medium containing 2% FBS or other medium containing d-ICD. The cells were synchronized with 1 mM thymidine (Sigma-Aldrich). Cells that migrated into the wound area were calculated as follows: ((0 h wound wide subtract 48 h wound wide)/0 h wound wide).

### 4.6. Trans-Well Invasion Assay

The trans-well insert (8-mm pore; Merck Millipore, Billerica, MA, USA) was pre-coated with 20% matrigel (BD Bioscience, San Jose, CA, USA) for 30 min at 37 °C. A total of 2 × 10^5^ cells were seeded onto the upper chamber of the trans-well in serum-free media, while the bottom chamber of the trans-well was filled with complete medium with or without d-ICD. After 24 h of incubation, the cells were fixed with 10% formaldehyde for 30 min and stained with crystal violet solution. Invasive cells were quantified using ImageJ software.

### 4.7. Dual Luciferase Reporter Assay

First, 293T cells were seeded onto 96-well culture plates for 16 h and then transiently co-transfected with *ITGA1* promoters and PRL-TK and the relevant plasmid. After 48 h of incubation, *Renilla* and *firefly* luciferase activity was determined according to the manufacturer’s instructions (Promega).

### 4.8. Chromatin Immunoprecipitation Assay (ChIP)

Chromatin immunoprecipitation assays (ChIP) were performed as previously described [33].

### 4.9. Cell Apoptosis Analysis

Cells were digested, and washed twice with 1× PBS, then resuspended in binding buffer to a concentration of 1 × 10^6^ cells/mL. Cell suspensions (500 µL) were added to tubes, then added 5 uL of Annexin V FITC conjugate and 10 µL of Propidium Iodide Solution (both available from Sigma-Aldrich) and incubated at room temperature for 10 min and protected from light. Fluorescence of the cells were analyzed by flow cytometry.

### 4.10. Cell Proliferation Assays

For MTT assays, 1000–3000 cells per well were plated on 96-well plates and incubated for 16 h. Then, 100 μL of MTT reagent (5 mg/mL, Sigma-Aldrich) was added to each well and incubated for 4 h at 37 °C. The optical density (OD) value was recorded at a dual wavelength (570 nm) every day for 7 days. For colony formation assays, 1000–3000 cells per well were plated on 6-well plates and cultured for 2 weeks, then fixed with 10% formaldehyde for 30 min at 37 °C. The cells were stained with Giemsa solution. Finally, the cell colonies were quantified.

### 4.11. Statistical Analysis

All data were presented as the mean ± standard deviation (SD). Differences between groups were assessed using Student’s *t*-test. Differences were considered statistically significant at *p* < 0.05. Analyses were performed using Prism 5.0 (GraphPad, San Diego, CA, USA).

## 5. Conclusions

In summary, the present study is the first study to demonstrate that d-ICD inhibits the migration and invasion ability of HCC cells in vitro. Furthermore, *ITGA1* is an important target of d-ICD and is involved in the effects of d-ICD on migration and invasion in HCC cells. Moreover, d-ICD downregulates *ITGA1* expression may partly through suppressing the expression of *E2F1* in HCC cells. Taken together, our observations help to better understand the effect and mechanism of d-ICD treatment in HCC cells as well as the function of *ITGA1* in HCC. More valuable findings regarding d-ICD in HCC treatment should be uncovered in future studies.

## Supplementary Materials

Supplementary materials can be found at www.mdpi.com/1422-0067/18/3/514/s1.
