# Supplementary material for: Derivate Isocorydine (d-ICD) Suppresses Migration and Invasion of Hepatocellular Carcinoma Cell by Downregulating ITGA1 Expression
Source: Int J Mol Sci. 2017 Feb 27;18(3):514. doi: 10.3390/ijms18030514 (PMC5372530; doi:10.3390/ijms18030514)
Supplement: Supplementary file 1 [file ijms-18-00514-s001.pdf]

Supplementary Materials: Derivate Isocorydine (d-ICD) Suppresses Migration and Invasion of Hepatocellular Carcinoma Cell by Downregulating ITGA1 Expression

Xiaoqin Liu, Hua Tian, Hong Li, Chao Ge, Fangyu Zhao, Ming Yao and Jinjun Li

Table S1. Antibodies used in this study.

Antibody	Host	Dilution	Company
ITGA1	Goat polyclonal	1:400 for WB	R&D system
E2F1	Mouse mAb IgG2a	1:100 for WB	Santa Cruz
ITGB1	Mouse mAb IgG2a	1:200 for WB	Santa Cruz
Cleaved-PARP	Rabbit IgG2a	1:500 for WB	Cell Signalling
β -actin	Mouse mAb	1:10000 for WB	Sigma

WB: Western blotting.

Table S2. The PCR primers for RT-PCR.

Name	Primer Sequence
ITGA1	Forward: 5′- CAGCAAGAAAGGAGGCATTC -3′<br>Reverse: 5′- TTTCCTCGGTTATAGCTGCC -3′
E2F1	Forward: 5′- CCGTGGACTCTTCGGAGAAC -3′<br>Reverse: 5′- ATCCCACCTACGGTCTCCTC -3′
GAPDH	Forward: 5′- AGAAGGCTGGGGCTCATTTG -3′<br>Reverse: 5′- AGGGGCCATCCACAGTCTTC -3′
CHIP	Forward: 5′- CGGAGAACGAGATCACCCTCT -3′<br>Reverse: 5′- CTCTAAATGCGCTCCACGC -3′

Table S3. The sequences of ITGA1 ShRNA.

Name	Primer Sequence
ShNC	TTCTCCGAACGTGTCACGT
Sh-ITGA1-1	Forward:5′-CCGGCCCTAGATTCACTAAGACAAA<br>CTCGAGTTTGTCTTAGTGAATCTAGGGTTTTTG -3′<br>Reverse:5′-AATTCAAAAACCCTAGATTCACTAA<br>GACAAACTCGAGTTTGTCTTAGTGAATCTAGGG -3′
Sh-ITGA1-2	Forward:5′-CCGGCCCTCGAAACACAACCTTTAACTCGAGTTAAAGGTTG<br>TGTTTCGAGGGTTTTTG -3′<br>Reverse:5′-AATTCAAAAACCCTCGAAACACAACCTTTAACTCGAGTT<br>AAAGGTTGTGTTTCGAGGG -3′
SiRNA-E2F1-1	Forward:5′- GGACCUGGAAACUGACCAUTT-3′<br>Reverse:5′-AUGGUCAGUUUCCAGGUCCTT-3′
SiRNA-E2F1-2	Forward:5′- GCAUCUAUGACAUCACCAATT-3′<br>Reverse:5′- UUGGUGAUGUCAUAGAUGCTT-3′

Table S4. Primers for vector constructs.

Name	Primer Sequence
E2F1:	Forward: 5′- GCGGATCCATGGCCTTGGCCGGGG -3′<br>Reverse: 5′- CCGGAATTCTCAGAAATCCAGGGGGGTGAGG -3′
ITGA1-promoter (P1-1822bp)	Forward: 5′- GGGGTACCATGGCCTTCAACCTCCTTGGGT -3′<br>Reverse: 5′- CCGCTCGAGGTGCTGCCTCACTCCTACT -3′
ITGA1-promoter (P2-830bp)	Forward: 5′- GGGGTACC CCACCCTTTAACCACGAAT -3′<br>Reverse: 5′- CCGCTCGAGAGCAGGCGACAGCGACCCCTGG -3′
ITGA1-promoter (P3-350bp)	Forward: 5′- GGGGTACCTCCTGCCCTGCGAACCAG -3′<br>Reverse: 5′- CCGCTCGAGGTGCTGCCTCACTCCTACT -3′
ITGA1-promoter (PM-mutant)	Forward: 5′- GGGGTACC CCACCCTTTAACCACGAAT -3′<br>Reverse: 5′- CCGCTCGAGCTATAGCCGCAGTTACG
	TGTTTAGGCTAAAGTCCACGGGCTGTAGAAGC -3′

Table S5. ITGA1 promoter sequence.

>ITGA1 promoter

-1521 ATGGCCTTCAACCTCCTTGGGTCTGCCCACCCCATTTTTGACGCTCCTTAA
-1470 ACTTCTTCAACTTTTGTCTTCTCCAGCTTTTAGTTGCTGTGATCCTCTATCT
-1418 GGCAATGTATCCAGTGAACCCTGTGGGCTTCTTCCTTCCTGTCCCTCGTG
-1367 TTACCTGTTCTGTGCTACCTGTGTTGCGTTCTGCTCAACGAACCCTCCTTT
-1317 GGGTGGCACTCTTGTCCTGCCTAGCCCTAGTTCTGCTAGAGATGCTCAAT
-1265 TAGTTGTTGAGTTGAAGAATTGAATTGAACTGTTTCCCTCAGATTCCTCTAA
-1213 ACACAAATCCACTTATTCTTTCTGTCTTTGAAATTCTCAAAACCGCTAAGAC
-1162 ACAGCTATTTTGAAAATAGCCAGCATTGGTTGAAGCTAGGGTGAATATGGC
-1112 TTTTGACTCAGAGAGGTTGTTCAACTCACCAGGAAAGGGAAGCATTGCAA
-1060 GTTCCTAAGCACTTCTGGACTCTTAATGCATTTTCACTCGTGAAAGTTTCAA
-1009 GCAAGAAAAAAATAAAGTAGTGCCCCGAGTTCTTTATTAGATTCAATTACC
-958 AGCGTACAAACATCCTAATTCACTTAAACAAAATAGCCCACACACATTCCA
-906 TTTCCAAGGAGGCTGCTTTTCTTCCTCTCTTCCCTTCTCGCTCTCCCTCTTA
-852 CTGTTTTTCCTCCGTCTTGCTTCTCCTTTTCTCCCCTTATCCTAGTTATCTC
-801 AAAGAAAAGCTTATCTGCAACCACCCTTTAACCACGAATTACCATAAACGA
-752 AGCAACCCCCCCACCCCCCCATAAGCGAAAGCAAAGCCCTCAGTGGAGC
-703 CTGTCGGGGTGGTGGGGACGCCCCCTTTCGTGCAAAACGACTTCACGGT
-654 GAATTTCAAGTGTCCGCAGGGGATGGAAGGGGGGATTCCAGATCCTCGC
-604 ATTTTCACAGACGGCCTAATCCCTCTGCGCAATGGACCCGGGAAAGCTGC
-555 CTCCCGTTAAGGGCGCCCAGGCACTGTCAAAGCGCCCTCCCTGCCCGGG
-507 CCGGGAGAAGTAAGGGGTGTGAAGGCGGAGCCACTGGGCTGGCAACAC
-457 CCTCCCGAAGGTCCAGCCCTTACCCGCCTTCCTACCACCTTAGGGGATTT
-407 GGCCCGGAGAACGAGATCACCCTCTCAATGAAAGGCAGATGTCCCTTTAA
E2F1 binding site
-357 GGTTTGCTTCTACAGCCCGTGGACTTTAGCCTAAACACGGACCCGCGAAG
-307 CTGGCTTTATTTGTCCATGTCTCGGACAGAGCCTGGGAAGCTGCCAGTGA
-258 GATTTCAGAGACCAAGAGCGCGAAGGGGCGGGCGATGTGGCAATCCGTC
-208 TGGGATGTGAAAAGCGTGGAGCGCATTTAGAGGAATTCGACGAAAACACA
-159 GGAAATCACTCCTCTCCCGCTCCTGGGCGCCGCTGCCACTGGGGCAGAG
-111 GACTGGGAACCGCGGCAGCGGGATAAGTGGCCCAGCCAGAGAGCGCAG
-63 CTCCCGCGCCCGGTCCTGCCCTGCGAACCAGCGCGGCCCCCTGGCGCT
Transcriptional initiation site
-15 GAGGCTGCTCCGGCCATGGCCCCTCGGCCCCGCGCCCGCCCAGGGGTC
+34 GCTGTCGCCTGCTGCTGGCTCCTCACTGGTGAGCGACTCGCTTTTCTCTG
+84 AGCATCTCCTGCTCGCGGGCTTGGGGCTTGGAGCGGGGAGGGAGGTCCT

+134 CAGAGCCATGGGCCAGATAGGAAGAGAGAGCGCCCATCCACTTTCGGGC
+182 ATTCCAGGTACTCAGGCCAGGCTACGGGGCAGGCAGGAGTTTCGCGTTC
+ +232 TGGTTTTGATATGAATTAGTTTTCGTGTCTCTCCAAAGTCCTTCACCCTAGG
+283 AGTAGGAGTGAGGCAGCAC

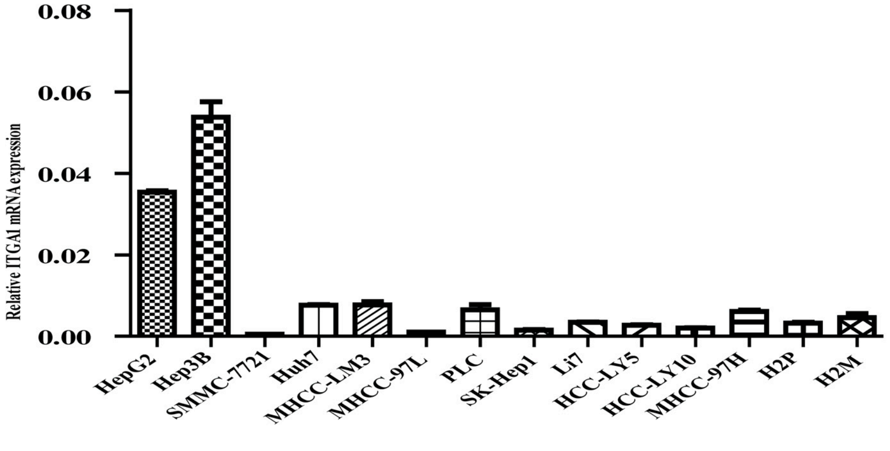

Figure S1. RT-PCR analyzed endogenous ITGA1 mRNA expression in various hepatocellular carcinoma (HCC) cell lines.

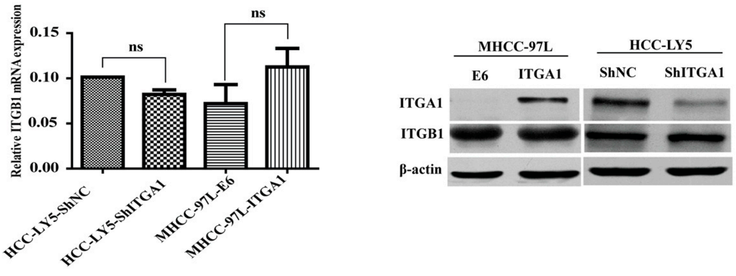

Figure S2. The mRNA and protein level of ITGB1 in ITGA1 overexpression HCC cells and ITGA1 silencing HCC cells were detected by RT-PCR and western blot. Results showed that the expression change of ITGA1 have no impact on ITGB1 in HCC cells. (“ns” indicates no statistical significance).

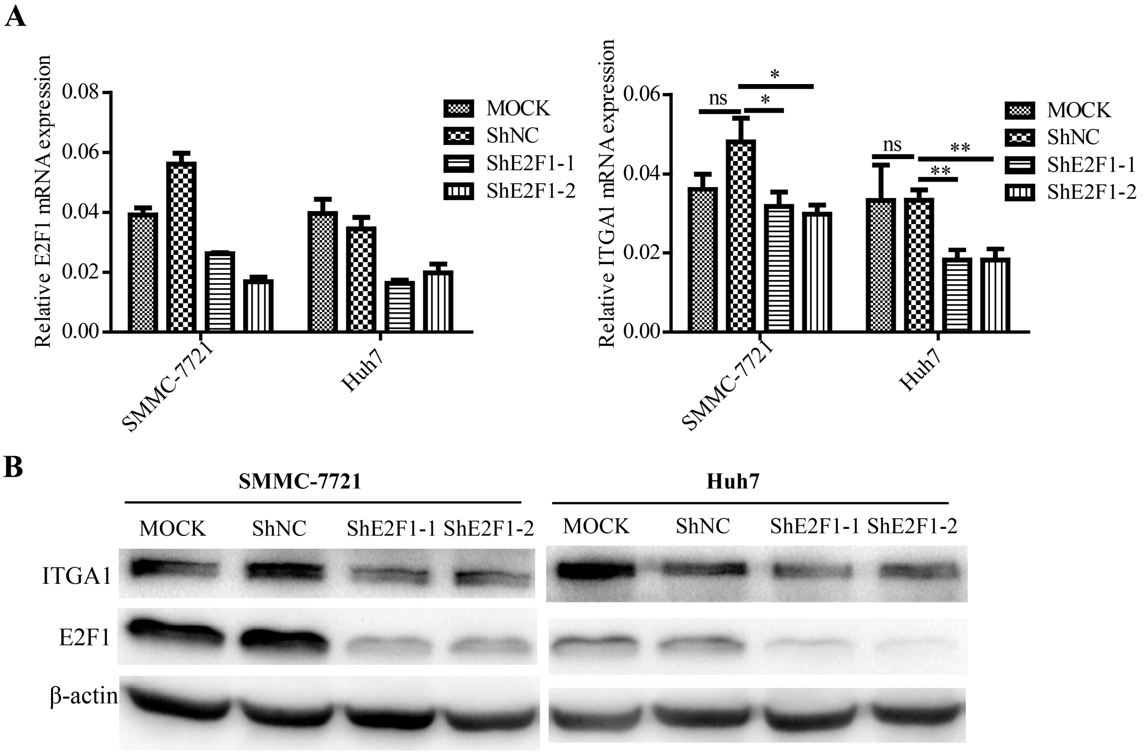

Figure S3. (**A**,**B**) RT-PCR and western blotting analyzed E2F1 and ITGA1 expression in HCC cells with transiently transfected E2F1 SiRNA. ("ns" means no significance, * $p < 0.05$, ** $p < 0.01$).

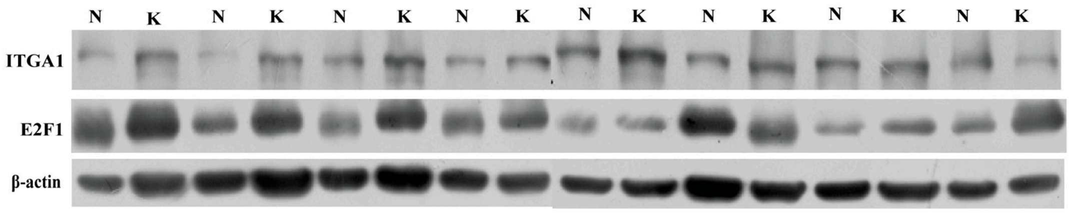

Figure S4. Western blot analyzed the expression of ITGA1 and E2F1 in HCC tissue. The result showed that the expression of ITGA1 positive related to the expression of E2F1 in HCC.

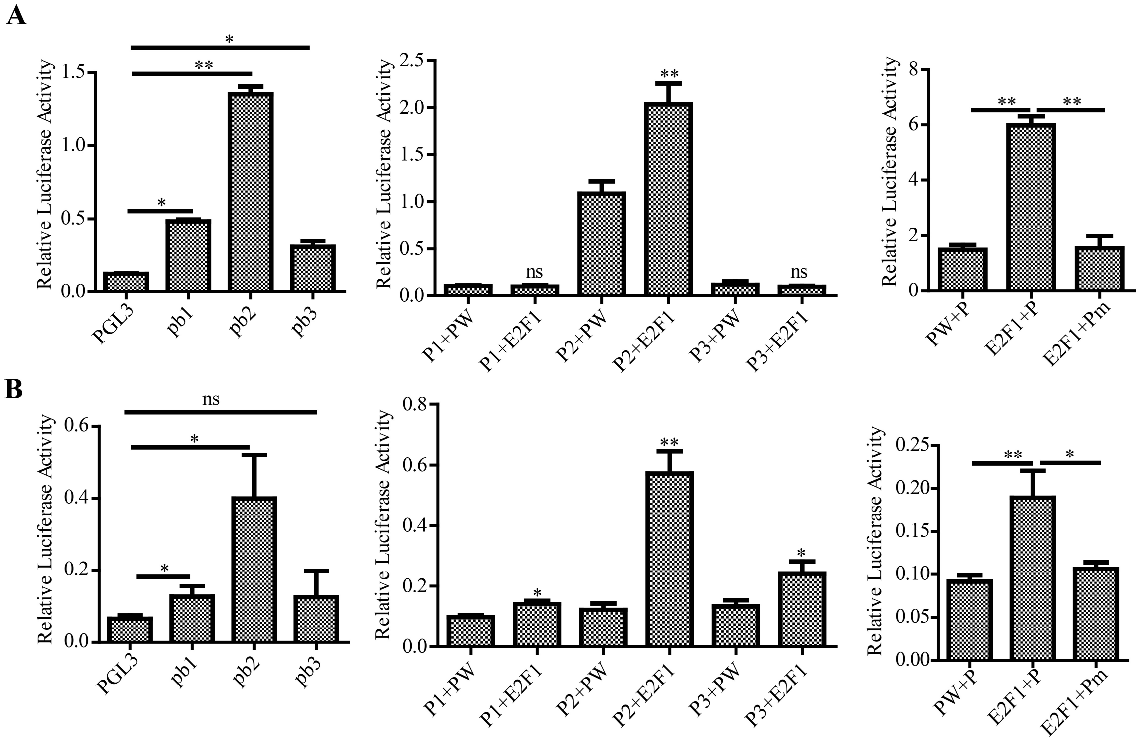

Figure S5. E2F1 binds to ITGA1 promoter in HCC cells. (**A**,**B**) Dual luciferase reporter gene studies were performed to detect the ITGA1 promoter and its truncated and mutant construct activity in SMMC-7721 and Huh7 cells. ("ns" means no significance , * $p < 0.05$, ** $p < 0.01$).

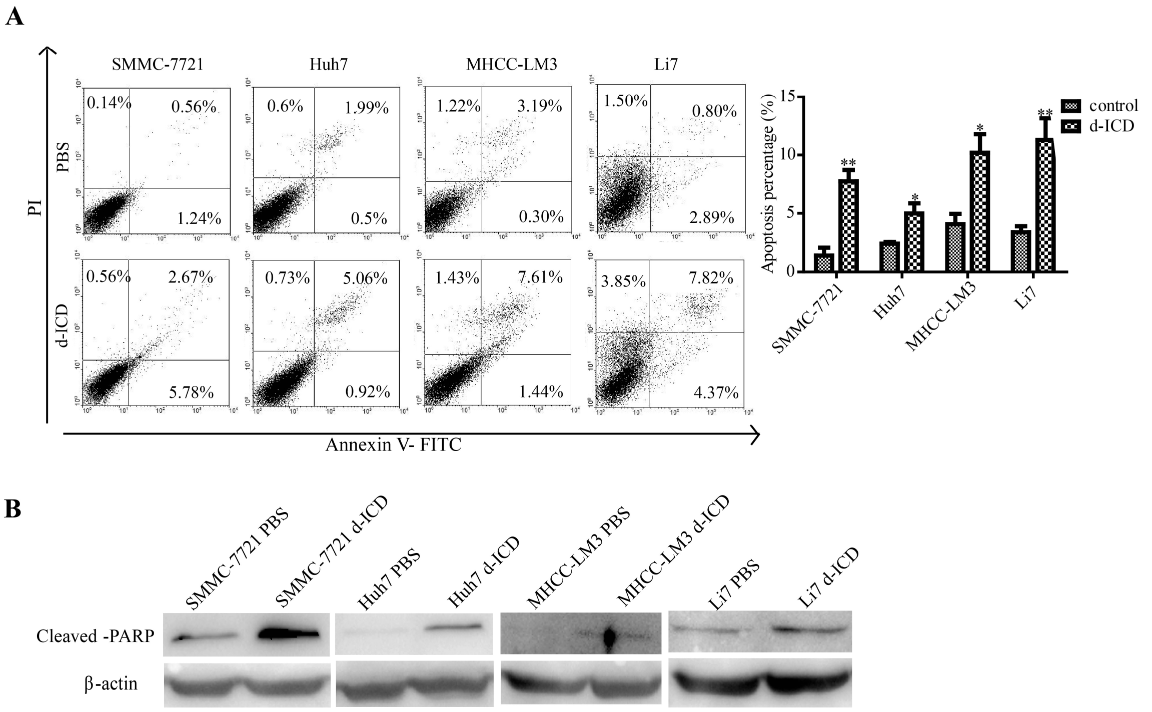

Figure S6. Derivate isocorydine (d-ICD) mildly induce HCC cell apoptosis during migration assay. (**A**) Following the treatment of SMMC-7721, Huh7, MHCC-LM3, Li7 cells with d-ICD for 48h, cells were stained by Annexin V-FITC and PI, and apoptosis cells were detected by FACS analysis. (**B**) Western blot analysis of cleaved PARP in SMMC-7721, Huh7, MHCC-LM3, and Li7 cells following d-ICD treatment 48h (* $p < 0.05$, ** $p < 0.01$).

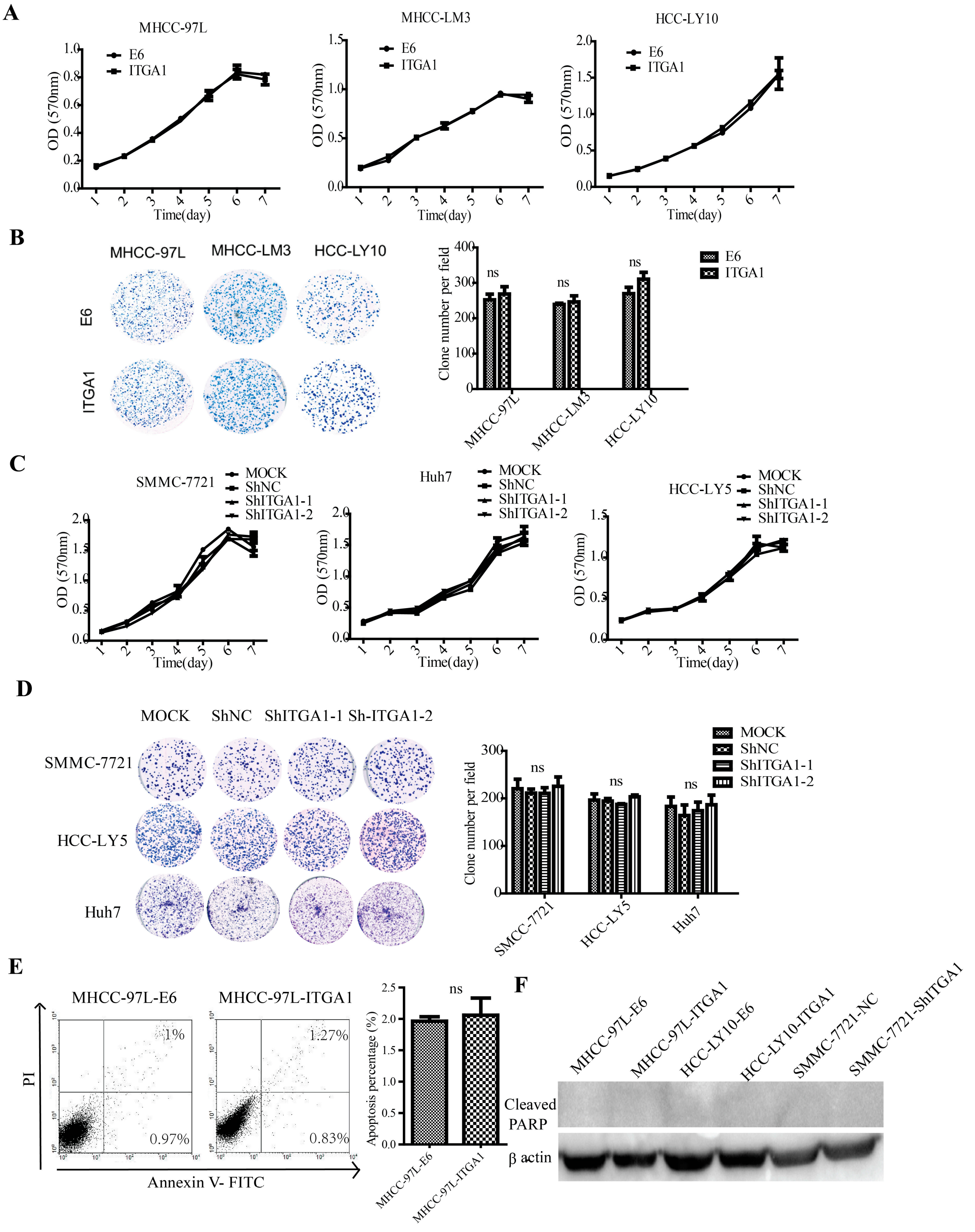

Figure S7. ITGA1 has no obvious effect on HCC cells proliferation or apoptosis in vitro. (**A**,**B**) MTT assay and clone formation assay were performed to analyze ITGA1 over-expression HCC cell proliferation ability, and the statistical analysis showed no significance between the two group. (**C**,**D**) MTT assay and clone formation assay were performed to analyze ITGA1 silencing-expression HCC cell proliferation ability, and the statistical analysis showed no significance between MOCK, ShNC, and ShITGA1 group. (**E**) Annexin v-FITC and PI double staining and FACS analyzed apoptosis cells in ITGA1 overexpression MHCC-97L cell and the control group cell, no difference was observed. (**F**) Western blot analysis of cleaved PARP in MHCC-97L and HCC-LY10 with ITGA1 overexpression and SMMC-7721 with ITGA1 silencing ("ns" means no significance).
